# Interstitial Perfusion Culture with Specific Soluble Factors Inhibits Type I Collagen Production from Human Osteoarthritic Chondrocytes in Clinical-Grade Collagen Sponges

**DOI:** 10.1371/journal.pone.0161479

**Published:** 2016-09-01

**Authors:** Nathalie Mayer, Silvia Lopa, Giuseppe Talò, Arianna B. Lovati, Marielle Pasdeloup, Stefania A. Riboldi, Matteo Moretti, Frédéric Mallein-Gerin

**Affiliations:** 1 Laboratory of Tissue Biology and Therapeutic Engineering, CNRS UMR 5305, Université Claude Bernard-Lyon 1 and University of Lyon, Institute for Biology and Chemistry of Proteins, Lyon, France; 2 Cell and Tissue Engineering Laboratory, IRCCS Galeazzi Orthopaedic Institute, Milan, Italy; 3 Bioengineering Laboratories S.r.l., Meda, Italy; 4 Regenerative Medicine Technologies Lab, Ente Ospedaliero Cantonale (EOC), Lugano, Switzerland; 5 Swiss Institute of Regenerative Medicine (SIRM), Lugano, Switzerland; 6 Fondazione Cardiocentro Ticino, Lugano, Switzerland; Ecole normale superieure de Lyon, FRANCE

## Abstract

Articular cartilage has poor healing ability and cartilage injuries often evolve to osteoarthritis. Cell-based strategies aiming to engineer cartilaginous tissue through the combination of biocompatible scaffolds and articular chondrocytes represent an alternative to standard surgical techniques. In this context, perfusion bioreactors have been introduced to enhance cellular access to oxygen and nutrients, hence overcoming the limitations of static culture and improving matrix deposition. Here, we combined an optimized cocktail of soluble factors, the BIT (BMP-2, Insulin, Thyroxin), and clinical-grade collagen sponges with a bidirectional perfusion bioreactor, namely the oscillating perfusion bioreactor (OPB), to engineer *in vitro* articular cartilage by human articular chondrocytes (HACs) obtained from osteoarthritic patients. After amplification, HACs were seeded and cultivated in collagen sponges either in static or dynamic conditions. Chondrocyte phenotype and the nature of the matrix synthesized by HACs were assessed using western blotting and immunohistochemistry analyses. Finally, the stability of the cartilaginous tissue produced by HACs was evaluated *in vivo* by subcutaneous implantation in nude mice. Our results showed that perfusion improved the distribution and quality of cartilaginous matrix deposited within the sponges, compared to static conditions. Specifically, dynamic culture in the OPB, in combination with the BIT cocktail, resulted in the homogeneous production of extracellular matrix rich in type II collagen. Remarkably, the production of type I collagen, a marker of fibrous tissues, was also inhibited, indicating that the association of the OPB with the BIT cocktail limits fibrocartilage formation, favoring the reconstruction of hyaline cartilage.

## Introduction

The incidence of degenerative and traumatic cartilage lesions is increasing due to the global population ageing and to the more intensive practice of sport activities. Articular cartilage is an avascular tissue with poor intrinsic healing ability. Consequently, cartilage injuries are irreversible and often evolve to osteoarthritis (OA), causing pain and disability. Several surgical approaches, such as mosaicplasty or microfractures, have been developed to repair damaged cartilage to avoid or at least delay joint replacement. However, these treatments remain unsatisfactory due to the occurrence of type I collagen-rich fibrocartilage [1, 2] which has inferior biomechanical properties than type II collagen-rich hyaline cartilage.

Articular cartilage, containing a single type of resident cells, is a good candidate for cell-based therapies and tissue engineering approaches. Autologous chondrocyte implantation (ACI) [3] is now performed worldwide to treat articular cartilage focal lesions. In this procedure, human articular chondrocytes (HACs) are isolated from a small cartilage biopsy and amplified *in vitro*, before being re-implanted back in the patient. However, during *in vitro* expansion, HACs undergo a dedifferentiation process characterized by the loss of type II collagen expression in favor of type I collagen. This process is a major determinant in the formation of type I collagen-rich fibrocartilage following ACI [4–6]. Hence, to improve ACI clinical outcomes, research has been focused on the use of biomaterials and growth factors able to drive HAC redifferentiation and broaden ACI application to larger defects, such as early OA lesions.

We have previously investigated the reconstruction of cartilaginous matrix by HACs in collagen sponges [7]. We used a combination of soluble growth factors during HAC expansion (fibroblast growth factor (FGF)-2 and insulin, designated FI) and 3D culture in collagen sponges (bone morphogenetic protein (BMP)-2, insulin, and thyroxin T3, designated BIT), that had been optimized for human auricular chondrocytes in collagen gels [8]. We found that the FI-BIT combination allows high HAC proliferation and cartilaginous extracellular matrix (ECM) synthesis in collagen sponges [7]. However, we also showed that ECM was restricted to the scaffold periphery, suggesting impaired nutrition and growth factor delivery in the sponge core.

Perfusion bioreactors have been shown to enhance cell access to oxygen and nutrients and the homogeneity of neo-synthesized ECM in 3D scaffolds [9, 10]. We have previously shown that the association of dynamic culture in an oscillating perfusion bioreactor, namely the OPB [11], with specific growth factors enhanced cell differentiation in tissue-engineered cardiac grafts, indicating that perfusion increases growth factor efficacy [12]. Furthermore, we have used the OPB to engineer cartilaginous constructs, demonstrating increased total collagen content and improved matrix deposition upon dynamic culture [13].

These results prompted us to investigate the combination of perfusion culture in the OPB with the aforementioned protocol of amplification/redifferentiation [7] for the culture of osteoarthritic HACs in clinical-grade collagen sponges. Special attention was given to the neo-synthesized matrix through the analysis of type I and type II collagen deposition.

## Materials and Methods

### Cell isolation and expansion

HACs were isolated from macroscopically non-fibrillated zones of osteoarthritic joints from 10 donors (age range: 50–74) undergoing total hip replacement. The study was carried out in accordance with local ethics guidelines and with the approval of the Scientific Directorate of the IRCCS Galeazzi Orthopaedic Institute. Biopsies were collected during surgery as anonymous waste material after donors’ written informed consent. Chondrocytes were extracted through sequential enzymatic digestion with 0.2% trypsin (Sigma) and 0.15% bacterial collagenase A (Roche Applied Science), as previously described [14]. HACs were then seeded at 0.8×10^4^ cells/cm^2^ in complete medium composed of HG-DMEM/Ham’s F12 (mixed 1:1, Gibco) supplemented with 10% newborn calf serum (NCS, Hyclone), 50 μg/mL streptomycin (Panpharma) and 2 μg/mL amphotericin B (Bristol Myers Squibb). At this stage, cells were designated P0. After 36 h (P0-36h), complete medium was refreshed and further supplemented with 5 ng/mL FGF-2 (R&D Systems) and 5 μg/mL insulin (Umuline rapide, Lilly), namely the FI cocktail [7]. FI-supplemented complete medium was replaced three times a week. At about 90% confluence, cells were trypsinized and replated at the same density for another passage (P1). After each trypsinization, live cells were counted with Trypan blue exclusion. The entire monolayer expansion phase lasted 25–28 days for all the donors.

### Preparation of the collagen scaffolds

Redifferentiation experiments were performed in collagen sponges purchased by Symatèse Biomatériaux. These scaffolds are composed of native (90–95%) type I collagen and (5–10%) type III collagen and are prepared from calf skin. During the manufacturing process, the sponges were cross-linked by glutaraldehyde and sterilized with 25 kGy β-radiation to increase their stability (pore size ~100 μm, Symatèse Biomatériaux) [7]. The collagen sponges were then sized in our laboratory with a skin biopsy punch (0.1 cm^3^, 8 mm diameter x 2 mm height).

### Static culture

Passaged P1 HACs were suspended in complete medium and seeded into the sponges at 1.3×10^7^ cells/cm^3^ (1.3×10^6^ cells/sponge) as previously described [7]. The sponges were then placed in 6-well plates and incubated at 37°C for 2h. Complete medium supplemented with 50 μg/mL 2-phospho-L-ascorbic acid (Fluka) was added. After 48 h, medium was replaced with fresh complete medium containing 50 μg/mL 2-phospho-L-ascorbic acid supplemented or not with 200 ng/mL recombinant human BMP-2 (dibotermin-alpha, drug form of BMP-2, InductOs kit, Wyeth), 5 μg/mL insulin, and 100 nM thyroxin T3 (Sigma), namely the BIT cocktail [7]. Medium was replaced twice a week over a culture period of 21 days.

### Dynamic culture in the OPB

**Fig 1:**
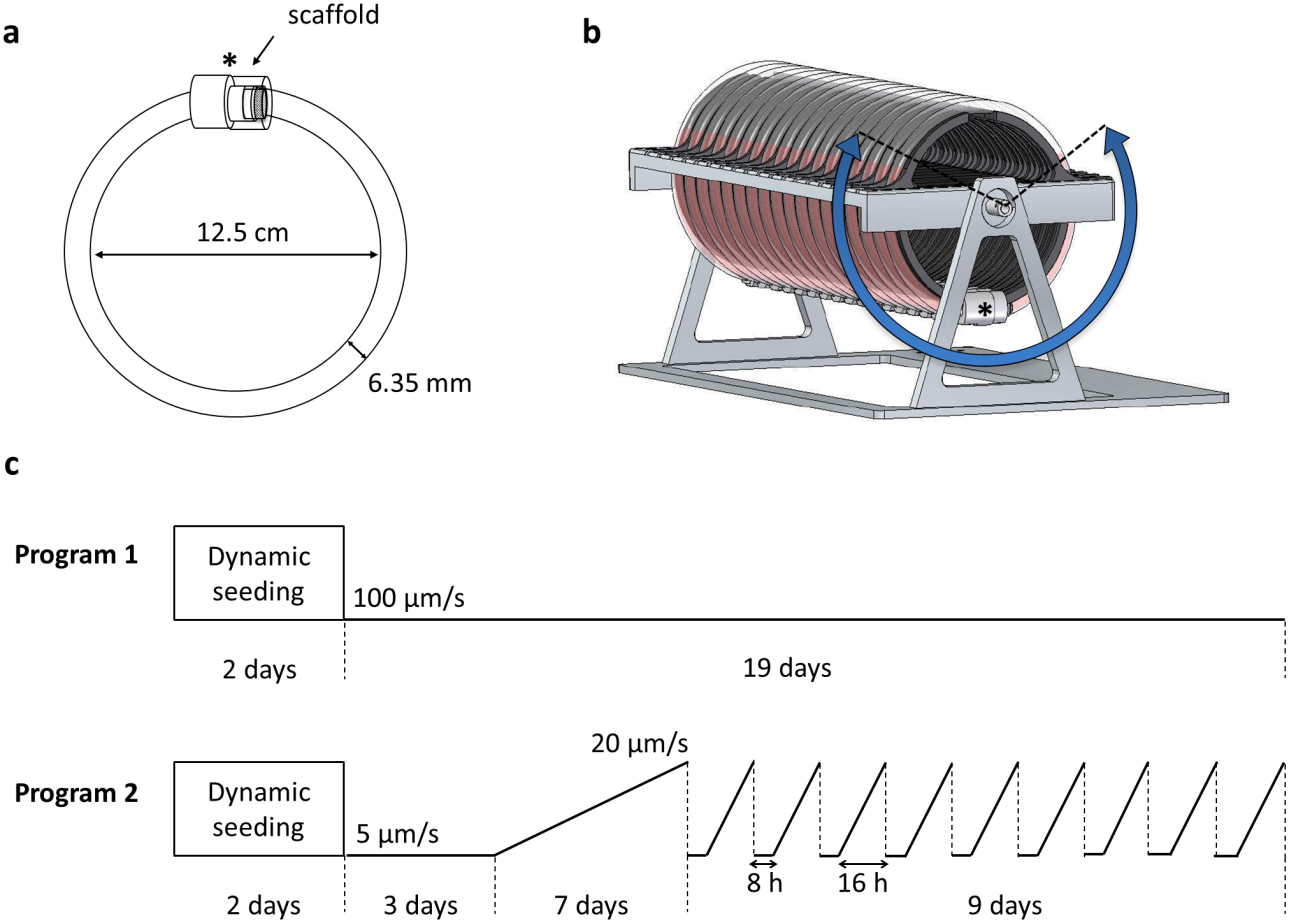
The Oscillating Perfusion Bioreactor (OPB). (a) The tissue culture unit is a closed loop of gas-permeable silicone tubing connected to a chamber (asterisk). A single collagen sponges is fixed within a scaffold holder inserted in the chamber to avoid fluid flow around the construct. (b) Eighteen independent closed-loop chambers are mounted on a motorized frame oscillating the chambers around their central axis in a pendulum-like motion. (c) Time sequences of the two perfusion programs used with the OPB.

The OPB was used as previously described [13]. Each collagen sponge was press-fitted into a scaffold holder that was then inserted in a chamber attached to a loop of gas-permeable silicone rubber tube (6.35 mm perfusion diameter, 1/32 inch wall thickness, Cole Parmer). Eighteen closed-loop chambers were mounted on 12.5 cm-diameter discs and then on an incubator-compatible motorized frame oscillating the chambers around their central axis (Fig 1). Interstitial fluid flow was ensured fixing the construct within the scaffold holder to avoid fluid flow around the construct.

Passaged P1 HACs were seeded at 1.3×10^7^ cells/cm^3^ (1.3×10^6^ cells/sponge). Specifically, 3 mL of cell suspension prepared in complete medium supplemented with 50 μg/mL 2-phospho-L-ascorbic acid were added to 7 mL of the same medium in each OPB chamber. The flow velocity was 1000 μm/s for 24 h, then 300 μm/s for additional 24 h. After 48 h, the medium in each chamber was replaced with 6 mL of complete medium containing 50 μg/mL 2-phospho-L-ascorbic acid, supplemented or not with BIT. Medium was replaced twice a week over a culture period of 21 days.

After cell seeding, two perfusion programs were applied (Fig 1c): in program 1, we applied a velocity of 100 μm/s during the whole culture. In program 2, we first applied a velocity of 5 μm/s for 72 h. Then, velocity was progressively increased up to 20 μm/s in 7 days and cycles of velocity variations were applied for the last 9 days (5 μm/s for 8 h followed by a progressive increase to 20 μm/s in 16 h).

### Gene expression analysis

Total RNA was isolated using the NucleoSpin RNA II kit (Macherey-Nagel). 100 ng RNA per sample were used for reverse transcription, as previously described [15]. Real-time PCR was performed in a Rotor-Gene Q cycler (Qiagen) using 10 μL Fast Start Universal SYBR Green Master (Roche), 4 μL cDNA (1:5dilution), 300 nM primers, and 4 μL water. Cycling conditions consisted of a denaturation step at 95°C for 2 min, 40 cycles of 95°C for 15 s and annealing and extension at 60°C for 30 s. Primer sequences were derived from literature [16]: *COL2A1* F: GGCAATAGCAGGTTCACGTACA, R: CGATAACAGTCTTGCCCCACTT; *COL1A1* F: CAGCCGCTTCACCTACAGC, R: TTTTGTATTCAATCACTGTCTTGCC; *GAPDH* F: ATGGGGAAGGTGAAGGTCG, R: TAAAAGCAGCCCTGGTGACC. Fluorescence thresholds (C_t_) were determined by the software (amplification efficiency range: 90%-110%). *GAPDH* C_t_ value was subtracted from the target C_t_ value to obtain ΔC_t_. Expression level was then calculated as 2^-ΔCt^ and expressed as the mean of duplicate samples.

### Antibodies

Antibodies were diluted in Tris-Buffered Saline-Tween (TBS-T) with 5% Blotting-Grade Blocker (BioRad) for Western Blot (WB) or in PBS containing 3% Bovine Serum Albumin (PBS-BSA) for immunohistochemistry (IHC). Polyclonal rabbit antibodies to human type II collagen (Ref 20211) were kindly provided by D.J. Hartmann [17] and used at 1:5000 for WB or 1:500 for IHC. Polyclonal rabbit antibodies to human type I collagen (Novotec, Ref. 20111) and human actin (Sigma, Ref. A5060) were used respectively at 1:5000 and 1:1000 for WB and the type I collagen antibodies were used at 1:1000 for IHC. Alkaline phosphatase-conjugated anti-rabbit or anti-mouse IgG (BioRad) and horseradish peroxidase (HRP)-conjugated anti-rabbit IgG secondary antibodies (Cell Signaling Technology) were used respectively at 1:5000 and 1:2000 for WB. For IHC, anti-rabbit (HRP)-linked antibodies (used undiluted) were from Dako (cat. K4002).

### Western Blot analysis

After 21 days of *in vitro* culture, collagen sponges were frozen in liquid nitrogen. After thawing, sponges were rinsed with PBS containing 2 mM EDTA and 0.2 mM phenylmethanesulfonylfluoride (PMSF), refrozen, powdered with a mortar and pestle, and boiled in Laemmli buffer for 5 minutes, as previously described [7]. For WB, equivalent sample volumes were separated by SDS-PAGE (4–12% gradient gels) and transferred to a polyvinylidene difluoride membrane (PVDF, Millipore) in CAPS buffer (N-cyclohexyl-3-aminopropanesulfonic acid, Sigma) supplemented with 7.5% methanol (90 min, 65V). After transfer, membranes were saturated in TBS-T containing 5% blocker for 1h at r.t., probed with primary antibodies either 1h at r.t. or overnight at 4°C, washed with TBS-T and incubated for 45 min at r.t. with secondary antibodies: alkaline phosphatase-conjugated IgG to detect type I and type II collagen or with HRP-conjugated IgG to detect actin. After washing, bound antibodies were detected on X-ray films using Immun-star AP or HRP chemiluminescent substrate (BioRad). Membranes were re-probed with antibodies after striping (Re-Blot Plus Strong, Chemicon).

For densitometric analysis, exposure times of X-ray films were optimized. The developed X-ray films were scanned and band intensities were determined using ImageJ software. The given values represent total collagen, namely the unprocessed chains (pro), the processing intermediates of the procollagen containing the aminopropeptide (pN) or the carboxypropeptide (pC) and the mature collagen chains, normalized to actin.

### Histological and immunohistochemical analysis

Histological analysis of collagen sponges was performed after 7, 14, and 21 days. Samples were rinsed in PBS and fixed for 24 h with 4% neutral-buffered formalin. After dehydration, samples were paraffin-embedded, sectioned at 5 μm, and stained with Mayer's hematoxylin and eosin (HE). To detect sulfated proteoglycans, sections were stained with Safranin-O in 0.1 M sodium acetate (pH 7.4) for 10 min.

For IHC, deparaffinized and rehydrated sections were digested with 0.5% hyaluronidase diluted in PBS-BSA for 1 h at r.t. to unmask the antigenic sites. Incubation with primary antibodies was carried out in PBS-BSA overnight at 4°C. After washing with PBS and PBS with 0.2% Tween-20, endogenous peroxidase activity was inhibited by treatment with 0.5% aqueous H_2_O_2_ in PBS-BSA. Then, HRP-conjugated secondary antibodies were applied for 45 min at r.t. Finally, sections were revealed with diaminobenzidine and counterstained with HE. Images were acquired with a DM750 microscope (Leica) with an integrated color camera (ICC50 HD) using the Las Ez software (Leica).

### In vivo subcutaneous implantation

After 21 days of *in vitro* static or dynamic culture, the constructs were subcutaneously implanted in the back of athymic mice to evaluate the maintenance of the redifferentiated phenotype of HACs and the stability of the neo-synthesized matrix, as previously reported by several studies [18–20]. The *in vivo* study was approved by the Mario Negri Institute for Pharmacological Research (IRFMN) Animal Care and Use Committee (IACUC). Animals and their care were handled in compliance with institutional guidelines, as defined in national (Law 116/92, Authorization n.19/2008-A, Italian Ministry of Health, March 6, 2008) and international laws and policies (EEC Council Directive 86/609, OJ L 358. 1, December 12, 1987; Standards for the Care and Use of Laboratory Animals-UCLA, U.S. National Research Council, Statement of Compliance A5023-01, November 6, 1998). The animals were regularly monitored by a certified veterinarian responsible for general animal health and welfare supervision, experimental protocols and procedure revision. All surgeries were performed under general anesthesia and all efforts were made to minimize suffering.

Six female athymic mice (six weeks old) were obtained from Harlan^®^ and maintained in the Animal Care Facilities of IRFMN, under pathogen-free conditions with food and water ad libitum. Surgical procedures were performed as described in previous studies [21, 22]. Briefly, animals were anesthetized by intraperitoneal injection of ketamine chloride (80 mg/kg, Imalgene, Merial) and medetomidine hydrochloride (1 mg/kg, Domitor, Pfizer) and surgeries were performed under a laminar flow hood in sterile conditions. Four unconnected subcutaneous pockets were created on the dorsum of each mouse by blunt dissection through cranial and caudal skin incisions. One construct was inserted in each pocket, then the skin was sutured with #4–0 Monocryl thread (Ethicon). Constructs from 3 donors were implanted (donors 8–10). For each donor, constructs cultured in static and dynamic conditions were implanted in the same mouse. After 6 weeks, the mice were euthanized by CO_2_ inhalation. At explantation, all constructs were dissected from the mice and processed for IHC.

### Statistical analysis

Quantitative differences in collagen production between experimental groups were analyzed using the non-parametric Mann-Whitney U-test. A p-value *p* < 0.05 was considered significant. The number of HAC donors used for each experiment/analysis is noted in the figure legends as “n”.

## Results

### Topography of the neo-synthesized matrix

**Fig 2:**
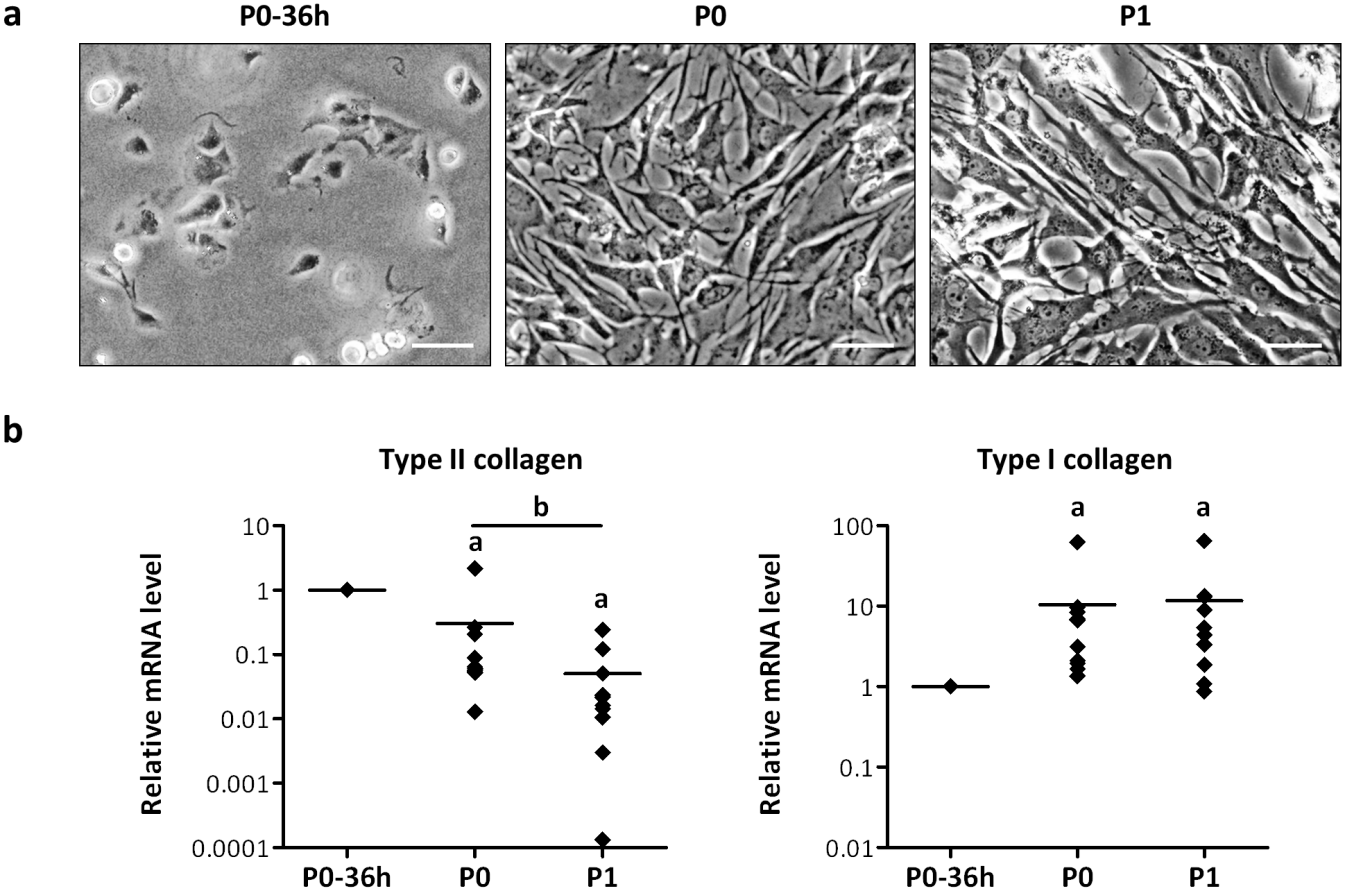
HACs undergo dedifferentiation during the amplification phase. (a) Representative phase-contrast micrographs showing changes in cell morphology during HAC expansion in monolayer (scale bars 50 μm). (b) Transcriptional levels of type I and type II collagen measured by Real-time PCR. Data were normalized on *GAPDH* using the 2^-ΔCt^ method, expressed relatively to data obtained for P0 HACs36 hours after isolation (P0-36h, reference value = 1) and reported as single data points with mean in the dot plots (n = 10, a: *p* < 0.05, significant effects versus P0-36h HACs; b: *p* < 0.05, significant effect versus P0 HACs).

After isolation, HACs were amplified in monolayer in the presence of FI. During this step, HACs lost their spherical shape and acquired a fibroblastic shape (Fig 2a). A marked decrease in type II collagen in favor of type I collagen expression was also observed during the amplification step (Fig 2b), indicating that HACs underwent dedifferentiation.

**Fig 3:**
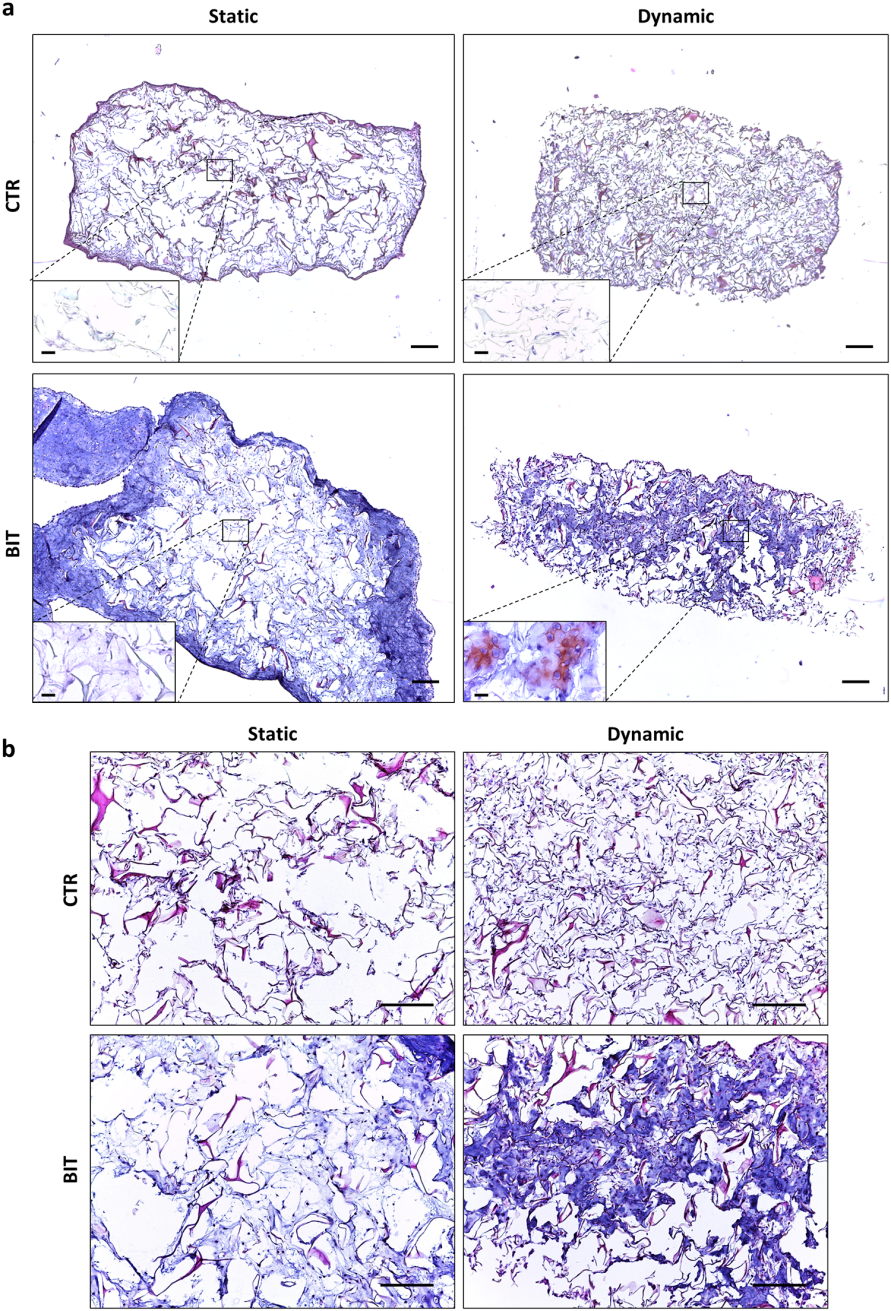
The combination of interstitial perfusion and BIT improves ECM neo-synthesis and distribution. (a) Representative low-magnification pictures showing cell and ECM distribution in collagen sponges cultured for 21 days in static or dynamic conditions (program 1, n = 4) in the absence (CTR) or in the presence of BIT (HE staining, scale bars 100 μm). Inserts in the left low corner show the Safranin O staining in the scaffold core (scale bars 10 μm). (b) High-magnification pictures showing cell and matrix distribution in the scaffold core (HE staining, scale bars 100 μm).

The dedifferentiated HACs were cultured in collagen sponges for 21 days in either static or dynamic conditions. In the absence of BIT, no matrix deposition was evidenced in both conditions (Fig 3a). When BIT was added in static conditions, peripheral deposition of ECM was observed, but no matrix was detected in the sponge core. Conversely, in the presence of BIT under perfusion, we observed that neo-synthesized ECM was homogeneously distributed throughout the scaffold. Besides, Safranin-O staining revealed a superior presence of sulfated proteoglycans in the core of dynamic samples cultured with BIT compared to static samples (Fig 3a, inserts). Additionally, a close look into the sponge core indicated that dynamic conditions improved cell distribution throughout the sponges, compared to static conditions (Fig 3b).

### Characterization of the neo-synthesized matrix

**Fig 4:**
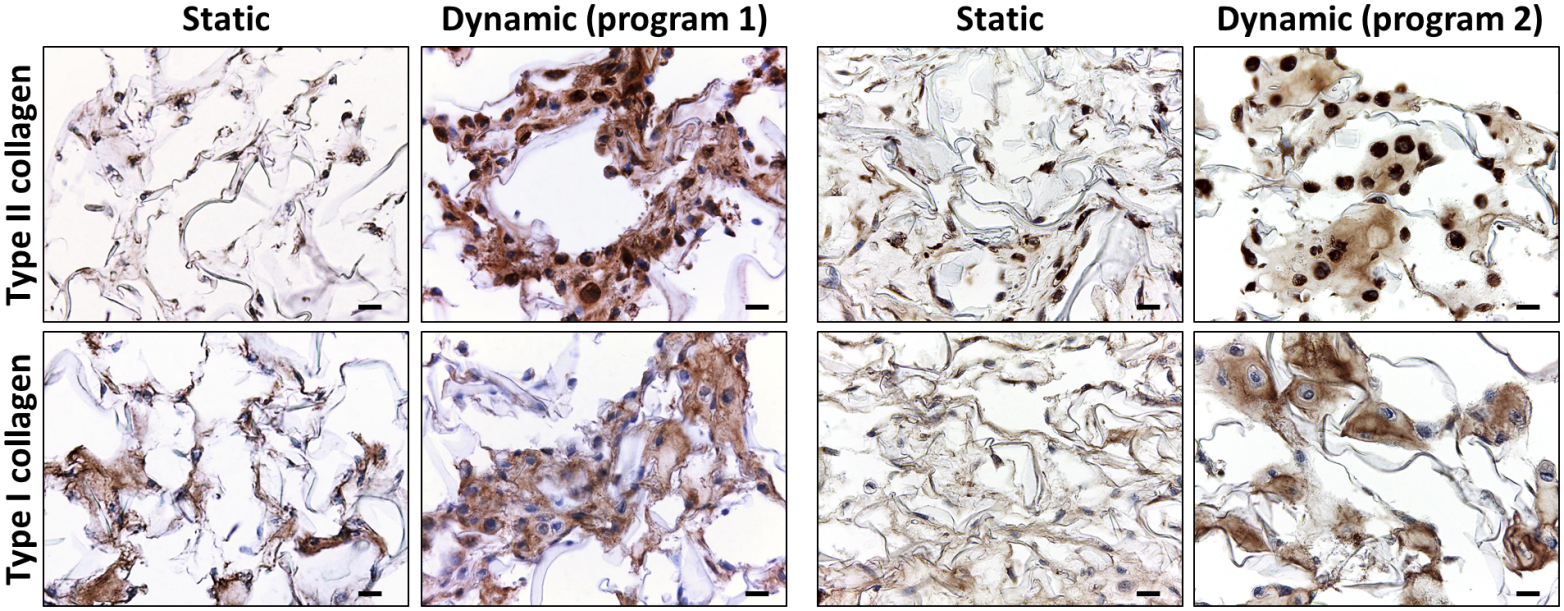
Interstitial perfusion enhances ECM production in the scaffold core. HACs were cultured for 21 days within collagen sponges in static or dynamic conditions using either program 1 (n = 4) or program 2 (n = 6) in the presence of BIT. Representative pictures show parallel sections of the scaffold core stained for type II and type I collagen by IHC (scale bars 10 μm).

To further characterize the ECM produced by HACs under the stimulation of BIT, we performed IHC for type II and type I collagen, markers of hyaline cartilage and fibrocartilage, respectively. To evaluate the impact of perfusion on HACs in the scaffold core, photographs of this region were taken at 21 days. In static conditions, HACs appeared isolated and exhibited a fibroblastic shape (Fig 4). By contrast, in dynamic conditions under either program 1 or 2, HACs exhibited a round morphology, typical of well-differentiated chondrocytes (Fig 4). In dynamic constructs, we observed zones with several HACs surrounded by neo-synthesized matrix immunoreactive for both type II and type I collagen (Fig 4).

**Fig 5:**
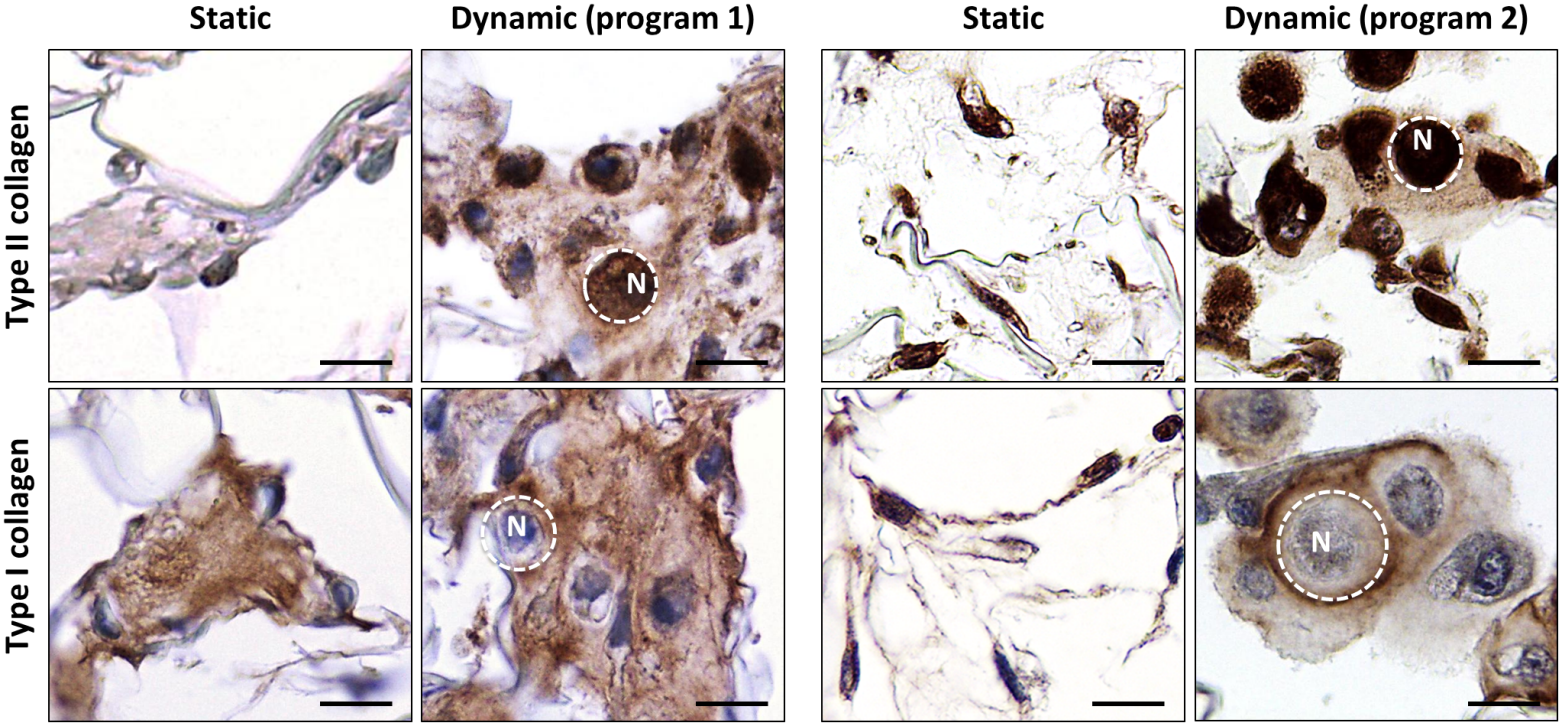
Cellular localization of type II and type I collagen after culture in static or dynamic conditions. Representative high magnification observations of HACs cultured for 21 days in static or dynamic conditions using either program 1 (n = 4) or program 2 (n = 6) in the presence of BIT (scale bars 10 μm). N: nucleus. The dotted lines indicate the cell border.

Interestingly, in perfused sponges we found type II collagen in cell cytoplasm, whereas type I collagen was detected in ECM but not in cell cytoplasm (Fig 5). Conversely, sponges in static conditions displayed type I collagen in both ECM and cell cytoplasm (Fig 5). Similar results were obtained when using program 1 and 2, indicating that the two perfusion programs resulted in equivalent patterns of type I and type II collagen expression.

### Perfusion culture hampers type I collagen production

**Fig 6:**
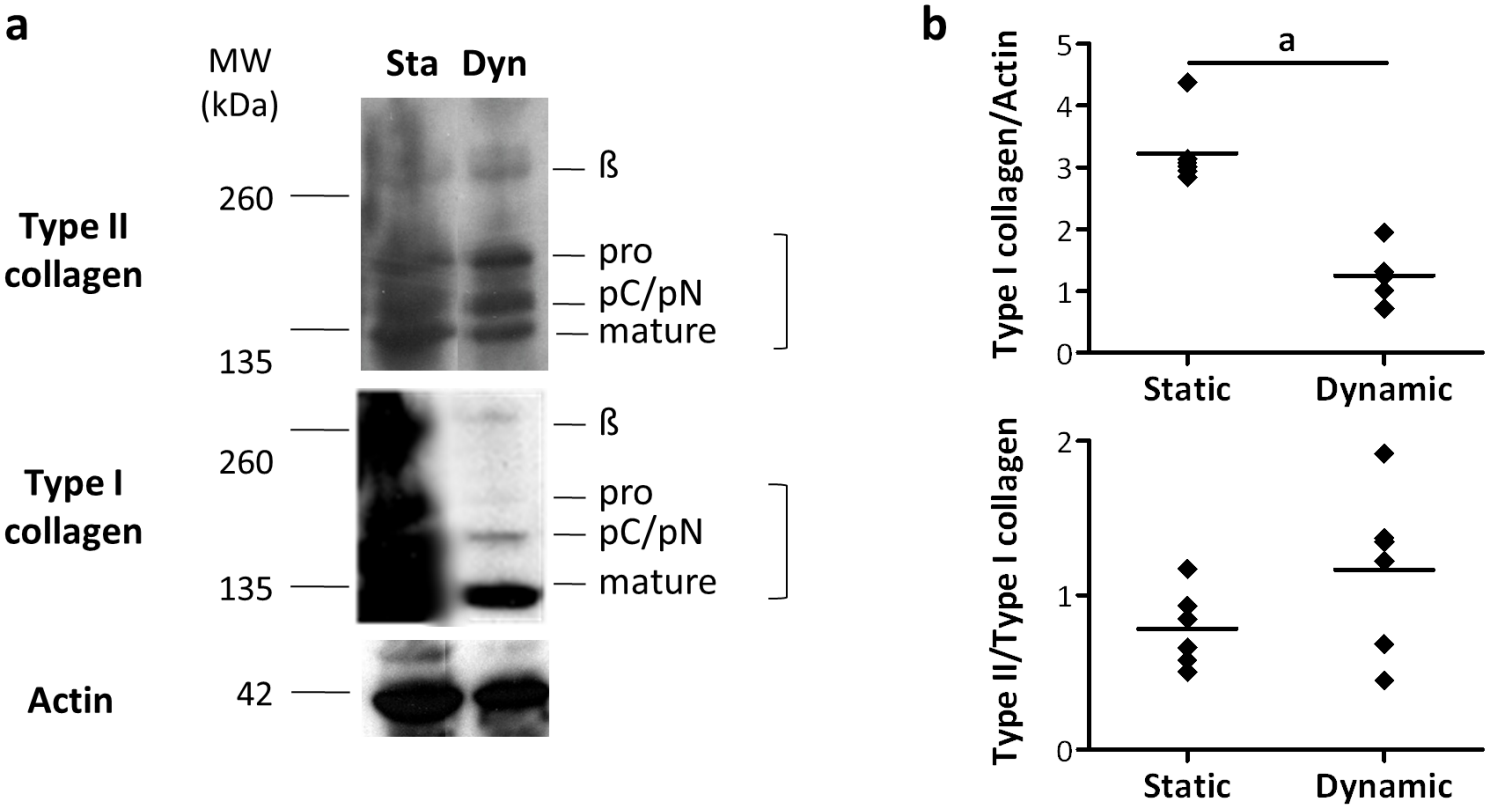
Type I collagen production is down-regulated by dynamic culture. (a) Representative WB analysis of type II and type I collagen after 21 days in static or dynamic conditions (program 2) in the presence of BIT. The positions of mature collagen chains (mature), unprocessed (pro) or processing intermediates of the procollagen containing the aminopropetide (pN) or the carboxypropetide (pC) are indicated. The upper band represents dimers (β) of collagen molecules. Actin bands are shown as a control for equivalent loading of the extracts. Please note that because type I collagen was more abundantly produced in static conditions, bands are smeared. (b) Quantification of type I collagen and of type II/type I collagen ratio through the densitometric analysis of WB data. Total type I and total type II collagen (i.e. the unprocessed chains, the processing intermediates of the procollagen, and the mature collagen chains) were normalized to actin. The values given in the dot plots represent single data points with mean (n = 6, a: *p* < 0.05).

To quantitatively determine the biochemical composition of the neo-synthesized matrix, WB analysis were performed on sponges cultured in static or dynamic conditions in the presence of BIT. Observation of total collagen production revealed that type II collagen, the typical component of the cartilage collagen fibrils, was synthesized in both conditions, whereas a lower amount of type I collagen was detected in the sponges cultured under perfusion (Fig 6a). The densitometric analysis of WB data confirmed a significantly lower production of type I collagen and showed a positive trend for type II/type I collagen ratio in sponges cultured in dynamic conditions (Fig 6b). WB analysis revealed that type II collagen was present as unprocessed, intermediate, and mature chains in sponges cultured in both static and dynamic conditions (Fig 6). In addition, in both conditions the presence of covalently cross-linked dimers of type II collagen indicated their possible organization into a fibrillar network. The presence of unprocessed, intermediate, and mature chains of type I collagen was clearly detected only in static constructs. Indeed, in sponges cultured under perfusion the presence of type I collagen was mainly restricted to mature chains, suggesting that HACs were no longer actively producing type I collagen. This result was in full concordance with IHC results showing that type II, but not type I collagen, was present in cell cytoplasm at the end of the perfusion culture.

**Fig 7:**
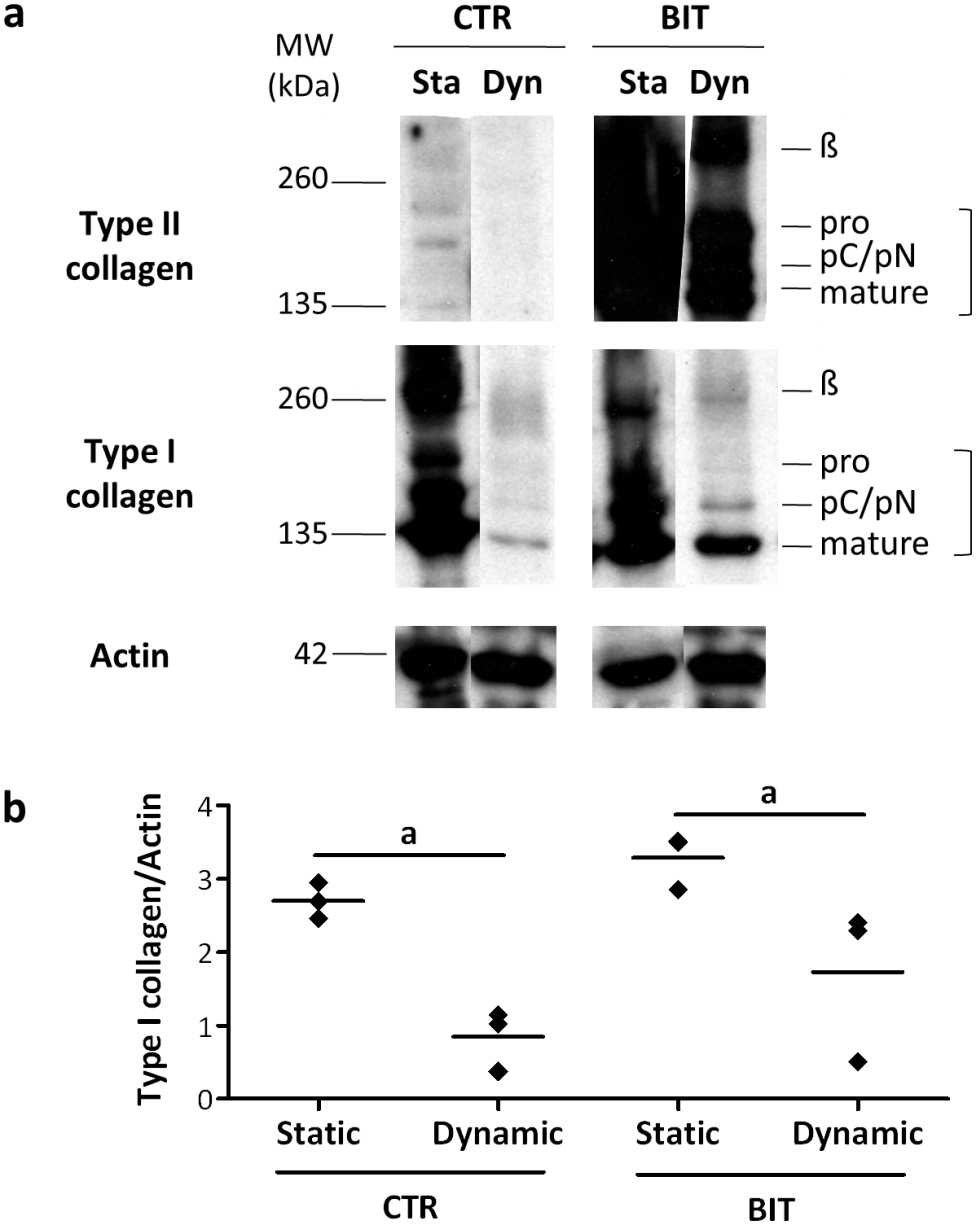
The down-regulation of type I collagen production in dynamic conditions is independent from the presence of BIT. (a) Representative WB analysis of type II and type I collagen after 21 days in static or dynamic conditions (program 1) in the absence (CTR) or in the presence of BIT. The positions of mature collagen chains (mature), unprocessed (pro) or processing intermediates of the procollagen containing the aminopropetide (pN) or the carboxypropetide (pC) are indicated. The upper band represents dimers (β) of collagen molecules. Actin bands are shown as a control for equivalent loading of the extracts. Please note that because type II collagen was relatively more abundantly produced in the presence of BIT, bands are smeared. (b) Quantification of type I collagen through the densitometric analysis of WB data. Total type I collagen (i.e. the unprocessed chains, the processing intermediates of the procollagen and the mature collagen chains) was normalized to actin. The values given in the dot plot represent single data points with mean (n = 3, a: *p* < 0.05).

Similar profiles of collagen synthesis were obtained when the perfusion program 1 was used (Fig 7). In particular, type II collagen was produced only upon culture with BIT, with no major differences between static and dynamic samples, while type I collagen was not strongly induced by BIT (Fig 7a). This observation was confirmed by the densitometric quantification of WB data (Fig 7b), showing that no significant differences were found in the production of type I collagen between CTR and BIT samples in both static and dynamic conditions. Importantly, both in the presence and in the absence of BIT, perfusion induced a strong decrease in type I collagen synthesis compared to static culture (Fig 7).

**Fig 8:**
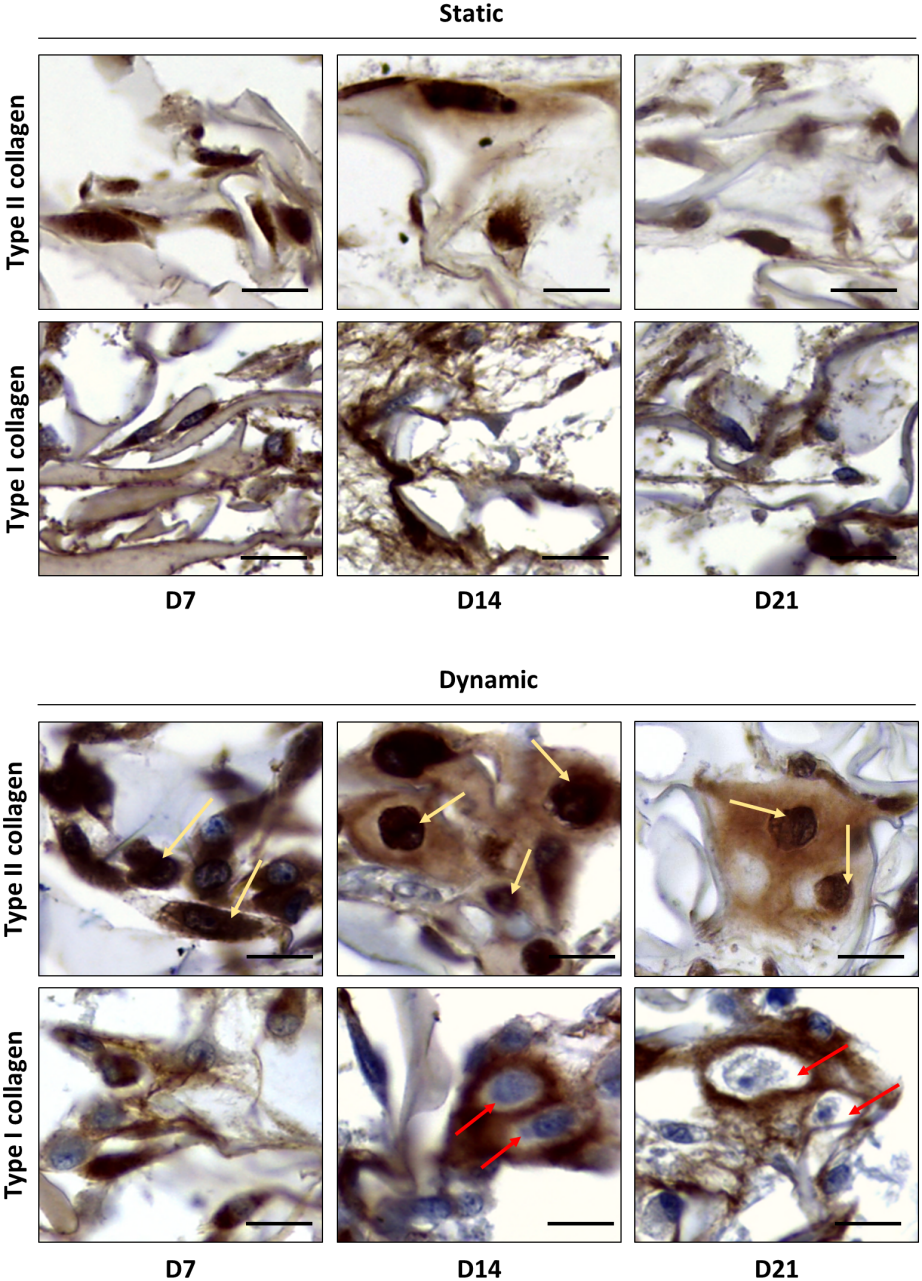
HACs interrupt type I collagen synthesis after 7 days of dynamic culture. Collagen sponges seeded with HACs were cultured in either static or dynamic conditions (program 1, n = 2) in the presence of BIT. At day 7 (D7), day 14 (D14), and day 21 (D21), IHC analyses were performed to localize type II and type I collagen. In dynamic conditions, note the absence of type I collagen in cell cytoplasm at day 14 and 21, as attested by the lack of brown staining (red arrows). Type II collagen synthesis was instead maintained during the whole culture, as demonstrated by cell cytoplasm staining (yellow arrows) from day 7 to 21 (scale bars 10 μm).

Altogether, our results indicated that a stop in type I collagen expression specifically occurred in dynamic conditions. Based on this, we examined cell morphology and collagen expression at different time points (7, 14, and 21 days). Special attention was given to the cells enclosed in the neo-synthesized ECM in the scaffold core. In static conditions, HACs maintained a fibroblastic shape from day 7 to day 21 (Fig 8). By contrast, HACs changed morphology during dynamic culture. Specifically, a fibroblastic shape was observed at day 7, whereas a round morphology was observed at days 14 and 21. IHC revealed the presence of type II collagen in cell cytoplasm at all the three time points in both dynamic and static conditions. In static conditions, the presence of type I collagen was detected both in the pericellular matrix and in cell cytoplasm at all the analyzed time points. Differently, in dynamic conditions, type I collagen was detected in the pericellular matrix from day 7 to day 21, but it was found in cell cytoplasm only until day 7 (Fig 8), indicating that at later time points HACs were no longer synthetizing type I collagen.

**Fig 9:**
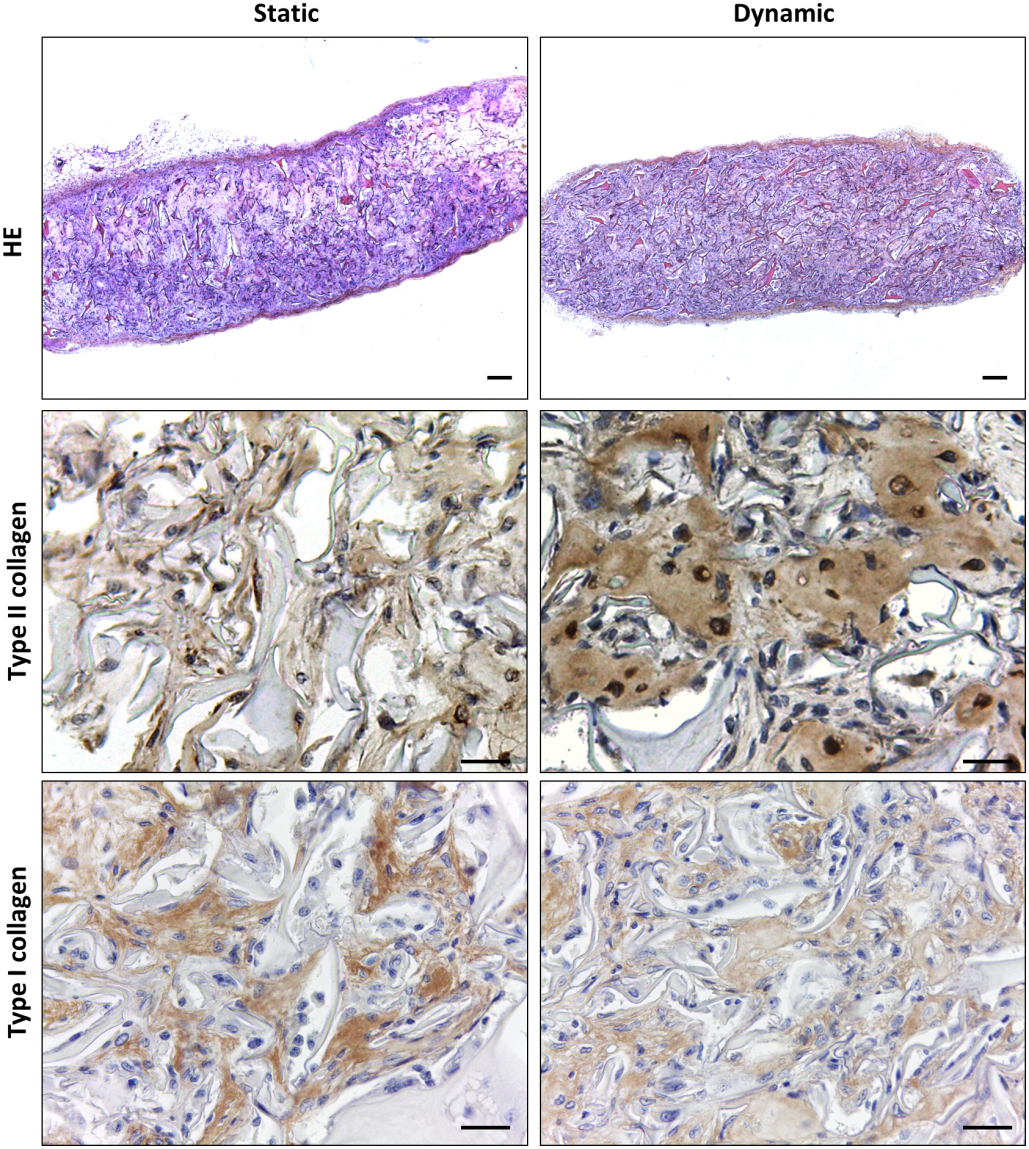
The engineered constructs remain stable after 6 weeks of subcutaneous implantation in nude mice. Collagen sponges seeded with HACs were cultured for 21 days in static or dynamic conditions (program 2, n = 3) in the presence of BIT and then implanted into the subcutaneous pockets of nude mice. Representative pictures showing HE staining of the sponges after 6 weeks *in vivo* are reported in the upper panel. The lower panels show parallel sections of the scaffold core stained for type II and type I collagen by IHC (upper panel: scale bars 100 μm; lower panel: scale bars 10 μm).

Finally, to evaluate the *in vivo* behavior of redifferentiated HACs and the stability of the neo-synthesized matrix built in the presence of BIT, the constructs were subcutaneously implanted in nude mice. After 6 weeks, the constructs maintained their structural integrity and were processed for IHC analysis. HE staining indicated that neo-synthesized ECM was more homogeneously distributed throughout the dynamic constructs in comparison with static constructs where ECM was preferentially deposited at the periphery (Fig 9). IHC analysis revealed accumulation of type II collagen in ECM and persistent synthesis in cell cytoplasm, whereas type I collagen was detected in ECM, but not in cell cytoplasm (Fig 9). These observations together indicated that the spatial distribution of ECM generated *in vitro* was maintained after 6 weeks *in vivo* and that chondrocytes were able to maintain their differentiation state after implantation.

## Discussion

We explored here the combined influence of a bidirectional perfusion bioreactor and of an optimized protocol for the redifferentiation of HACs in clinical-grade collagen sponges. It is well-known that amplification of HACs induces dedifferentiation, resulting in the progressive increase in type I collagen at the expense of type II collagen expression [23]. We have previously reported that FI, besides its proliferative effect on HACs, also stimulates HAC dedifferentiation [7]. In accordance with these previously published data, the morphological changes and gene expression data reported in the present study confirmed that HACs underwent dedifferentiation upon amplification with FI.

The OPB prototype takes into account important features for clinical translation, such as the possibility to perform a streamlined process including cell seeding and construct maturation in a closed-loop chamber, suitability to scale up and scale out, automation and ease of use [11]. Previous works have shown that perfusion culture enhances cell access to oxygen and nutrients and homogenous ECM deposition in 3D scaffolds [9, 24]. Here, we applied a relatively high flow velocity in the OPB during the cell seeding phase to improve seeding efficiency, and then two different perfusion programs. In program 1, a constant perfusion was applied using a flow velocity of 100 μm/s that has been previously shown to be suitable to obtain stable oxygen tension and homogeneous matrix deposition in 3D foam scaffolds [9]. Also the flow velocities used in program 2 were selected on the basis of previous studies. Pazzano *et al* [25] demonstrated that perfusion at 1 μm/s stimulated matrix neo-synthesis in 3D scaffolds. Other studies [26, 27] have shown that low flow velocity (10 μm/s and 7 μm/s) is required during the first days to protect early matrix deposition in porous scaffolds and that type II collagen and proteoglycan synthesis can be further enhanced slowly increasing the velocity from 7 to 19 μm/s. Here, we did not observe major differences between the two perfusion programs. Indeed, both programs improved cell distribution and the quality and homogeneity of the neo-synthesized matrix, compared to static culture. This was particularly evident in the sponge core, where neo-synthesis of type II collagen-rich ECM occurred in dynamic, but not in static conditions.

In this study, we paid particular attention to the nature of the collagen in the neo-synthesized matrix. WB analyses showed that type II collagen, a typical marker of native cartilage [28], was produced both in static and in dynamic conditions. Undoubtedly, the combination of the FI and BIT cocktails proved to be crucial to amplify and re-induce HACs to produce cartilage proteins, as previously shown [7]. Indeed, we found that perfusion in the presence of 10% NCS alone was not sufficient to promote cartilage matrix synthesis. Additionally, in static conditions, although BIT induced type II collagen synthesis, the neo-synthesized matrix was restricted to the scaffold periphery. The homogeneous distribution of cartilaginous matrix was found only in dynamic constructs cultured with BIT, demonstrating that the combination of perfusion and BIT substantially improved the homogeneous reconstruction of cartilage in collagen sponges.

We also demonstrated the beneficial influence of perfusion on the decrease in type I collagen synthesis. Indeed, our results revealed that HACs entered a redifferentiation program towards cartilage matrix production upon perfusion culture. Specifically, HACs initially maintained a dedifferentiated transient phenotype in dynamic conditions, as illustrated by their fibroblastic morphology at day 7. At this stage, most fibroblastic cells stained for both type I and type II collagen, suggesting the expression of a transient phenotype. At the same time, we found type I, but not type II collagen, in the matrix surrounding fibroblastic cells. These observations indicate that the dedifferentiated HACs were in an early redifferentiation phase during the first week of dynamic culture and that they started to redifferentiate only afterwards. This was confirmed by observations at day 14 and day 21 showing round cells and the presence of type II collagen in both ECM and cell cytoplasm, indicating that type II collagen synthesis was sustained during perfusion culture. In contrast, the presence of type I collagen became restricted to the neo-synthesized matrix, indicating that HACs were no longer actively producing type I collagen. Accordingly, precursor forms of type I collagen were hardly detectable by WB after 21 days of perfusion. Altogether, these observations demonstrate that perfusion stimulates the construction of a neo-matrix that matures over time with type II collagen, at the expense of type I collagen. This result is particularly striking considering the pathological origin of the articular chondrocytes used in this study, since the ability of HAC from OA joints to redifferentiate has been found to be impaired in other culture conditions [29]. Our findings are consistent with other studies showing that perfusion inhibits type I collagen expression in human chondrocytes cultured in pellets [30] and in cartilage explants [31]. The molecular mechanisms underlying the inhibitory effect of perfusion on type I collagen in chondrocytes are still unknown. Nevertheless, it is known that perfusion exerts mechanical forces, such as fluid shear stress [24], and that the expression of several ECM proteins, including type I collagen, is sensitive to mechanical forces in cartilage [32]. Thus, we can hypothesize that the fluid flow perfused using the OPB may have mechanically stimulated the chondrocytes seeded within the collagen sponges leading to a reduction in type I collagen expression. The BIT cocktail has also been found to inhibit, to some extent, type I collagen expression in human dedifferentiated chondrocytes [8, 33, 34]. However, the strong decrease in type I collagen seen in the sponges cultivated without BIT in the OPB indicates that perfusion was the main determinant of the type I collagen inhibition observed in our study.

Although the stability of the neo-cartilage and of the chondrocyte phenotype observed after 6 weeks in the nude mouse model is very encouraging, pre-clinical trials with long-term implantation in cartilage lesions in larger animals are required to test mechanical properties and integration of the implant. Nevertheless, our results already demonstrate the value of a multi-factorial approach combining HACs from osteoarthritic joints, clinical-grade collagen scaffolds, BIT cocktail, and perfusion culture. This strategy allows to achieve a high degree of redifferentiation in dedifferentiated chondrocyte, not only by inducing the specific production and homogeneous distribution of hyaline matrix, but also by inhibiting type I collagen synthesis. This result is particularly relevant since it indicates that such a multi-factorial approach minimizes the risk of producing fibrocartilage, even when osteoarthritic chondrocytes are used. Remarkably, special efforts were made to develop an approach employing clinical-grade growth factors and scaffolds which, combined with the intrinsic features of the OPB and its compatibility with GMP requirements, can be easily translated into the clinical practice. Lastly, since articular cartilage is chronically hypoxic and we have shown that hypoxia combined with BMP-2 favors the redifferentiation of human chondrocytes cultured in monolayer [35] or in collagen sponges [36, 37], it would be pertinent to investigate if hypoxic conditions can further improve the outcomes of our multi-factorial approach.

## Supporting Information

S1 Fig ECM distribution and cellular localization of type II and type I collagen after culture in dynamic conditions (program 1). Pictures of the inner core of the scaffolds showing HE, type II collagen and type I collagen staining of HACs from 4 donors cultured for 21 days in dynamic conditions using program 1 (scale bars 10 μm). Red arrows indicate cellular areas negative for type I collagen, demonstrating that HACs cultured in dynamic conditions are not synthesizing this type of collagen.
(TIF)

S2 Fig ECM distribution and cellular localization of type II and type I collagen after culture in dynamic conditions (program 2). Pictures of the inner core of the scaffolds showing HE, type II collagen and type I collagen staining of HACs from 6 donors cultured for 21 days in dynamic conditions using program 2 (scale bars 10 μm). Red arrows indicate cellular areas negative for type I collagen, indicating that HACs cultured in dynamic conditions are not producing this type of collagen.
(TIF)
