# Supplementary figures and images for: Interstitial Perfusion Culture with Specific Soluble Factors Inhibits Type I Collagen Production from Human Osteoarthritic Chondrocytes in Clinical-Grade Collagen Sponges
Source: PLoS One. 2016 Sep 1;11(9):e0161479. doi: 10.1371/journal.pone.0161479 (PMC5008682; doi:10.1371/journal.pone.0161479)

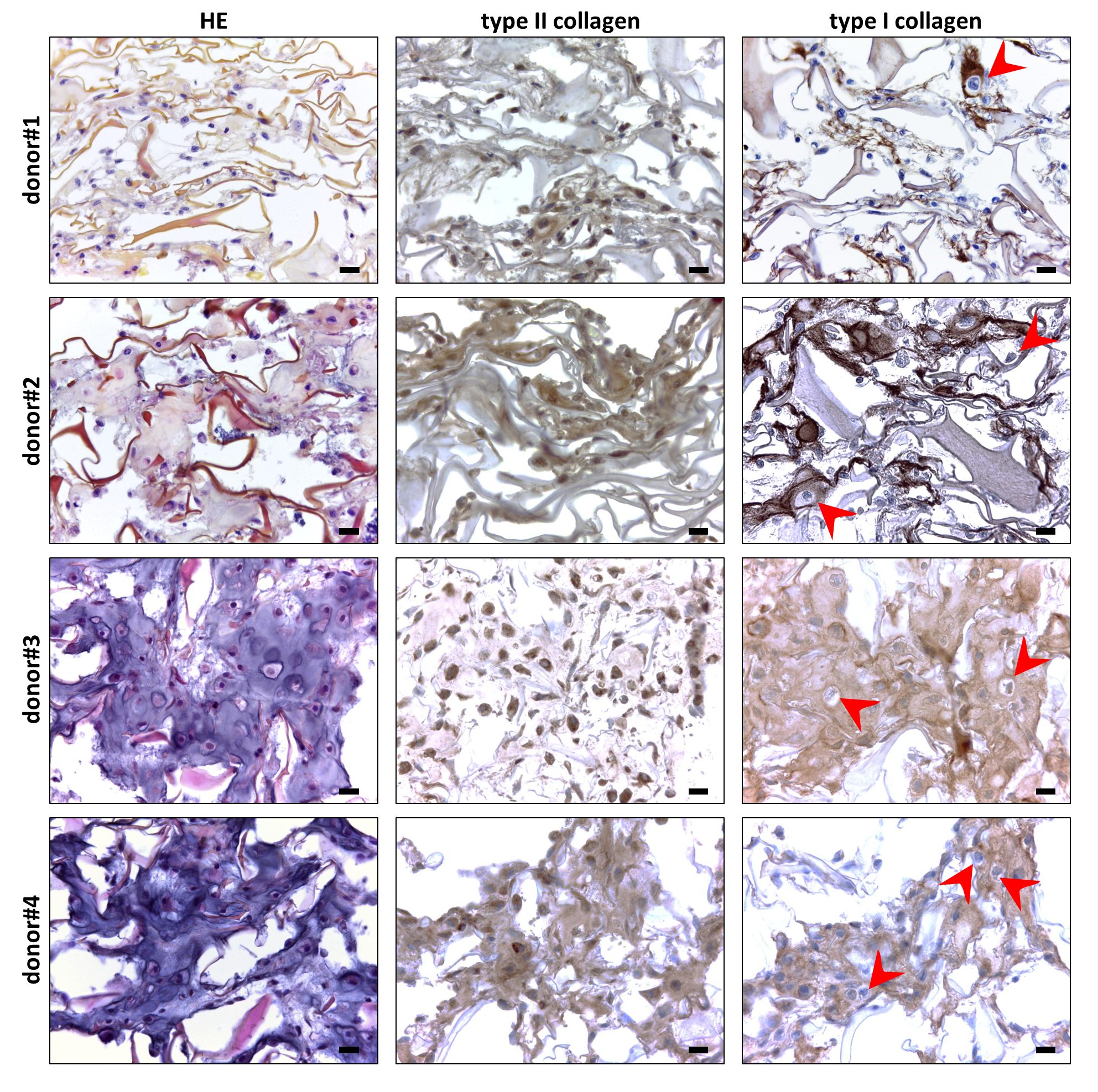

Supplement: S1 Fig — Pictures of the inner core of the scaffolds showing HE, type II collagen and type I collagen staining of HACs from 4 donors cultured for 21 days in dynamic conditions using program 1 (scale bars 10 μm). Red arrows indicate cellular areas negative for type I collagen, demonstrating that HACs cultured in dynamic conditions are not synthesizing this type of collagen. (TIF) [file pone.0161479.s001.tif]

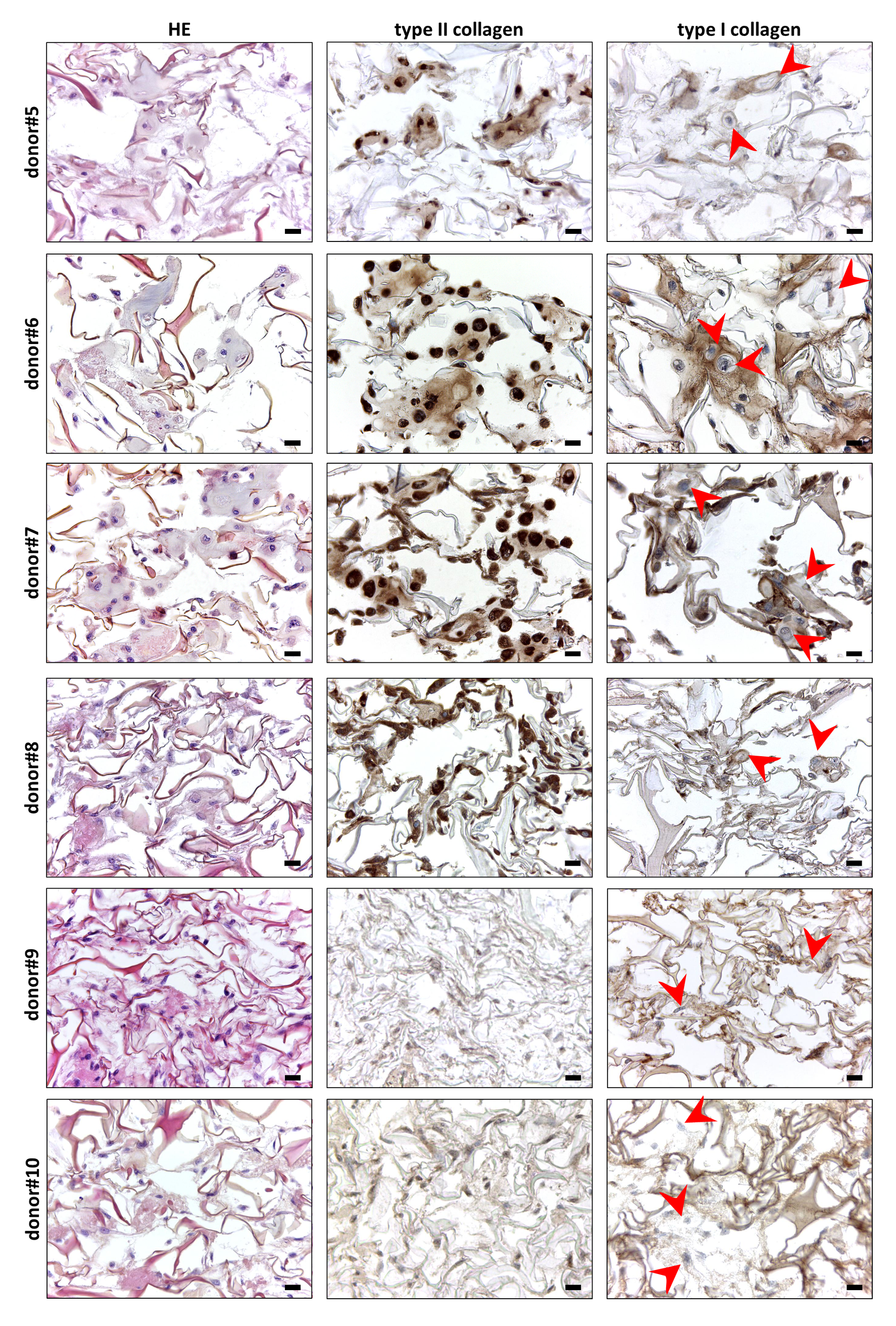

Supplement: S2 Fig — Pictures of the inner core of the scaffolds showing HE, type II collagen and type I collagen staining of HACs from 6 donors cultured for 21 days in dynamic conditions using program 2 (scale bars 10 μm). Red arrows indicate cellular areas negative for type I collagen, indicating that HACs cultured in dynamic conditions are not producing this type of collagen. (TIF) [file pone.0161479.s002.tif]
